# Phase Diagram-Based Sensing with Adaptive Waveform Design and Recurrent States Quantification for the Instantaneous Frequency Law Tracking

**DOI:** 10.3390/s19112434

**Published:** 2019-05-28

**Authors:** Angela Digulescu, Cornel Ioana, Alexandru Serbanescu

**Affiliations:** 1 Department of Communications and Military Electronic Systems, Military Technical Academy “Ferdinand I”, 050141 Bucharest, Romania; alexe1serbanescu@yahoo.com; 2 GIPSA-Lab, Université Grenoble Alpes, 38400 Saint Martin d’Hères, France; cornel.ioana@gipsa-lab.grenoble-inp.fr

**Keywords:** dynamic phenomena, phase space diagram, phase space lobe, instantaneous frequency law tracking, recurrence quantification analysis

## Abstract

Monitoring highly dynamic environments is a difficult task when the changes within the systems require high speed monitoring systems. An active sensing system has to solve the problem of overlapped responses coming from different parts of the surveyed environment. Thus, the need of a new representation space which separates the overlapped responses, is mandatory. This paper describes two new concepts for high speed active sensing systems. On the emitter side, we propose a phase-space-based waveform design that presents a unique shape in the phase space, which can be easily converted into a real signal. We call it phase space lobe. The instantaneous frequency (IF) law of the emitted signal is found inside the time series. The main advantage of this new concept is its capability to generate several distinct signals, non-orthogonal in the time/frequency domain but orthogonal within the representation space, namely the phase diagram. On the receiver side, the IF law information is estimated in the phase diagram representation domain by quantifying the recurrent states of the system. This waveform design technique gives the possibility to develop the high speed sensing methods, adapted for monitoring complex dynamic phenomena In our paper, as an applicative context, we consider the problem of estimating the time of flight in an dynamic acoustic environment. In this context, we show through experimental trials that our approach provides three times more accurate estimation of time of flight than spectrogram based technique. This very good accuracy comes from the capability of our approach to generate separable IF law components as well as from the quantification in phase diagram, both of them being the key element of our approach for high speed sensing.

## 1. Motivation

Time-frequency analysis is the natural way to characterize any non-stationary signal. A fundamental parameter of any non-stationary signal is the instantaneous frequency of each component. Generating separable components in time-frequency domain is a general aim when we look for sensing complex environment, characterized by multi-path channels. We will present further an interesting solution to this problem, based on the phase diagram analysis.

The instantaneous frequency (IF) law estimation is also a current topic, usually subjected to tracking in time-frequency domain, especially in the case of complex time-frequency content. The concept of phase diagram representation of a signal along with the recurrence quantification analysis (RQA) (related to the concept of nonlinear dynamic systems) allow tracking the IF law of a received signal. This provides a new method for the estimation of the IF law evolution in time. The main advantage of this approach is the possibility to use the phase diagram representation in order to highlight the signal’s phase continuity, useful for an accurate IF law tracking.

The initial idea of separable IF law in phase diagram domain has been presented in Digulescu’s Ph.D. thesis [1] and, in this paper, we propose a unified approach for high speed sensing. The idea proposed in this thesis is then combined with the approach of IF law estimation in phase diagram, presented separately in [2]. The presentation of global approach of high speed sensing is therefore the main motivation for this paper. The ideas mentioned before are integrated in the transmitter and receiver parts, respectively, and the experimental results prove the capability of this approach for high speed accurate sensing.

## 2. Introduction

The use of wide band signals with complex frequency modulations is nowadays a common topic in various applications such as acoustic sensing, radar, communications. The adaptive waveform concept is more and more used in an attempt to improve the sensing capabilities in terms of resolution and robustness. We will show in this paper how the phase diagram representation provides a very interesting solution, through defining a new way of designing separable waveforms. The waveforms defined in the phase diagram domain are the first stage of the high sensing technique addressed in this paper.

At the receiving level, the signals’ processing is reduced to tracking the IF law. The natural signals [3] generated by sources such as underwater mammals, the signals issued from dynamic configurations and radar signals represent just a few of the applications which justify the high interest in the characterization of signals with complex non-linear time-frequency structures. The characterization of such signals is often realized by the estimation of the IF law of the time-frequency components of the signal.

Indeed, characterizing signals with non-linear time-frequency component is a complex task that is always a current research topic for signal processing community. The main IF law estimation techniques are based on the tracking of the time-frequency maxima points extracted from time-frequency distributions such as spectrogram or quadratic time-frequency representations [4]. Generally, the most energetic points of the time-frequency distributions are merged in order to form the IF law of each component and, for this purposes, different time-frequency representations can be used: Wigner distribution [5], multi-window short time Fourier transform [6], pseudo-Wigner-Ville distribution [7], polynomial Wigner-Ville distribution [8]. For instance, different techniques have been proposed for the optimization of IF law estimation from time-frequency distribution based on Viterbi methodology [9,10,11,12].

The performances of all these techniques depend essentially on the quality of the initial time-frequency distributions. The appropriate setup of these distributions is often subject to a pre-analysis of the signal and, usually, requires a step-by-step optimization procedure.

An alternative to these approaches is proposed in this paper, as a joint concept based on the phase diagram properties exploited by the RQA, using the time-frequency continuity, a helpful property especially in the case of crossing and/or close time-frequency structures. Our method comes with a new approach based on the phase diagram representation and on the recurrence quantification analysis concept [13,14,15,16]. The phase diagram concept comes from the nonlinear dynamic systems theory and provides new ways for the signal representation.

Although the classical concept of RQA is usually related to the concept of recurrence plots (RP), our approach is based on the direct quantification of the phase diagram vectors. In this manner, we aim to disregard the method’s dependency on the type of chosen distance for the RP computation. Our approach exploits the equivalent of the angular distance for the RP proposed in [17,18] and it has the advantage that it skips the computation resources required by the distance matrix together with the “trained eye”-reliance for the quantification of the obtained RP.

Starting with the fact that a system characterized by a sine wave has an ellipse shape trajectory in the phase diagram, our approach explores the properties of such system which returns to a previously visited state, namely into a recurrent state. We are able to develop the concept of phase diagram RQA-based IF law tracking by quantifying the number of the system’s returns in these recurrent states using the evolution the position vector of phase diagram. In this way, we are able to quantify the dynamics of the system.

This quantification is obtained with the use of angular distance previously introduced in [14,15], which disregards the amplitudes of the signal and simply considers the position of the vectors in the phase space. A complete rotation of the vector in the phase space means that the angular distance is 2π, hereby the position vector has returned in a previously visited state. In this paper we will prove that the number of points between the current point of the phase diagram and its recurring point is inversely proportional to the fundamental frequency of the system at that time.

Based on these characteristics [15,16,17,18], we have developed a new method for the estimation of the frequency of a time-evolving signal.

**Figure 1:**
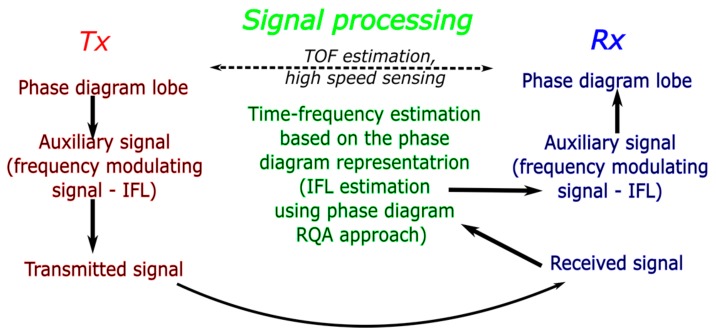
Paper organization: the transmission part (*Tx*) is presented in Section 3, the signal processing part based on the phase diagram properties is presented in Section 4 and the reception part (*Rx*) which includes the applications is presented in Section 5.

The paper is organized as follows (Figure 1): Section 3 shows the phase diagram representation and defines a new type of wide band signal for the emission suitable for the phase diagram representation. In Section 4, we will define the phase diagram RQA based IF law tracking using the signal’s properties. In addition, we will test the method’s robustness to noise. In Section 5, we illustrate the concept of high speed sensing based on phase diagram, suitable for the characterization of dynamic processes. The last part, Section 6, presents the conclusions and further developments of our work.

## 3. Phase Diagram Representation

### 3.1. Theoretical Aspects

This representation has the major advantage that does not use any model to analyze the data, unlike the classical methods [19,20], hereby it is a data driven technique.

The phase diagram concept starts with considering the following time series:

(1) $$x=\left\{x[1],x[2],\ldots ,x[N]\right\}$$

where *N* is the length of the time series.

This time series is represented in phase space, leading to the creation of the phase diagram. Its values become the coordinates of the *m*-dimensional space and, consequently, the vector sample is given by [13]:

(2) $$\vec{v_{\left[i\right]}}=\sum_{k=1}^{m}x[i+(k-1)d]\cdot \vec{e_{k}},\;\;\;\;i\in \{1,2,\ldots ,M\}$$

where *m* is the embedding dimension of the phase space, *d* is the delay chosen between the samples, $\vec{e_{k}}$ is the unit vector of the axis that defines the phase space where *k* position is the only non-zero component and M=N−(m−1)d. Usually, the delay is computed using the mutual information method [21,22,23,24,25,26] or the multi-lag phase-space analysis [17,18]. Furthermore, the embedding dimension is chosen using the false nearest neighbor method [17,18].

**Figure 2:**
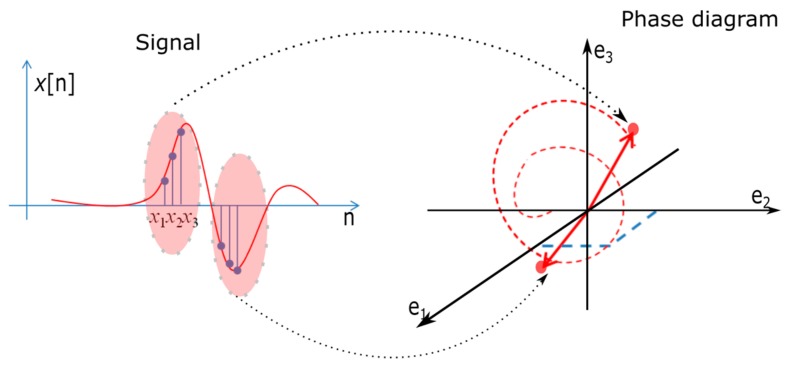
Illustration of the phase space trajectory computation.

Figure 2 illustrates the phase space construction algorithm. The coordinates of each point on the phase space trajectory are based on the values from the time series, namely signal’s samples.

### 3.2. Phase Diagram Lobes Used at Emission

In this part, we present the phase diagram property to contain separable trajectories, each one corresponding to a unique and separable signal from the others. This property can help us defining a set of non-orthogonal signals in time and frequency, but separable in phase diagram domain. This property is the origin of the phase-diagram-based modulation concept that is defined in this paper.

The starting point is the idea to design a pattern in the phase space with a unique evolution and, afterwards, to translate it in a 1D signal, into a one-to-one correspondence.

The simplest method to represent the waveform’s trajectory in shape of a lobe is to compute it with two vectors:

(3) $$\begin{array}{l} \vec{v_{n}}=a_{n}\cdot \vec{i}+b_{n}\cdot \vec{j}+c_{n}\cdot \vec{k},\;\;\\ n\in \{1,2\},\;\forall a_{n},b_{n},c_{n}>0\\ \vec{v_{n}}(a_{n},b_{n},c_{n}),\;\;n\in \{1,2\} \end{array}$$

Considering that phase space lobe is characterized by two points $P_{n},\;\;n\in \{1,2\}$, where $P_{n}\subset {\mathbb{R}}_{+}^{3}$ and the origin of the phase space O(0,0,0), we define the 1D signal, called auxiliary signal. The phase space representation of the auxiliary signal is given by the vectors $\vec{v_{1}}=\vec{OP_{1}}$ and $\vec{v_{2}}=\vec{P_{2}O}$, so the corresponding time series of the auxiliary signal is defined as follows:

(4) $$\begin{array}{l} \forall P_{n}(a_{n},b_{n},c_{n})\subset {\mathbb{R}}_{+}^{3},n\in \{1,2\},\;\;P_{1}\neq P_{2}\neq O,\;\;\exists \;x_{i_{n}},y_{j_{n}},z_{k_{n}}\in {\mathbb{R}}_{+},\\ \;\Rightarrow \mathrm{aux}=\{x_{1},\ldots ,x_{n_{1}},x_{n_{1}+1},\ldots ,x_{N},y_{1},\ldots ,y_{n_{2}},y_{n_{2}+1},\ldots ,y_{N},z_{1},\ldots ,z_{n_{3}},z_{n_{3}+1},\ldots ,z_{N}\} \end{array}$$

Applying some geometrical constraints to the phase space based lobe—width and continuity, the two vectors that create the phase space are described by:

(5) $$\begin{array}{l} \left\{ \begin{array}{l} \left\{ \begin{array}{l} x\left[i_{1}\right]=a_{1}\cdot t\left[i_{1}\right],\;\;i_{1}\in \{1,2,\ldots ,n_{1}\}\\ x\left[i_{2}\right]=-a_{2}\cdot t\left[i_{2}\right],\;\;i_{2}\in \{n_{1}+1,n_{1}+2,\ldots ,N\} \end{array}\right.\\ \left\{ \begin{array}{l} y\left[j_{1}\right]=b_{1}\cdot t\left[j_{1}\right],\;\;j_{1}\in \{1,2,\ldots ,n_{2}\}\\ y\left[j_{2}\right]=-b_{2}\cdot t\left[j_{2}\right],\;\;j_{2}\in \{n_{2}+1,n_{2}+2,\ldots ,N\}, \end{array}\right.\\ \left\{ \begin{array}{l} z\left[k_{1}\right]=c_{1}\cdot t\left[k_{1}\right],\;\;k_{1}\in \{1,2,\ldots ,n_{3}\}\;\\ z\left[k_{2}\right]=-c_{2}\cdot t\left[k_{2}\right],\;\;k_{2}\in \{n_{3}+1,n_{3}+2,\ldots ,N\} \end{array}\right. \end{array}\right.\\ a_{1,2},b_{1,2},c_{1,2}>0,\;\;\;n_{1,2,3}\in {\mathbb{N}}^{*},\;\;N\in {\mathbb{N}}^{*},\;\;t[n]=\frac{n}{f_{s}},\;\;n\in \{1,2,\ldots ,N\} \end{array}$$

where $f_{s}$ is the sampling frequency.

(6) $$\left\{\begin{array}{l} x[n_{1}]=x[n_{1}+1]\\ y[n_{2}]=y[n_{2}+1]\\ z[n_{3}]=z[n_{3}+1] \end{array}\right.$$

Using the continuity condition, Equation (6), and considering that the lobe returns to its original point, meaning that x[1]=x[N]=0, y[1]=y[N]=0, z[1]=z[N]=0, the relationship between the slopes of the two director vectors becomes:

(7) $$\left\{\begin{array}{l} a_{2}=a_{1}\cdot n_{1}/\left(N-n_{1}\right)\\ b_{2}=b_{1}\cdot n_{2}/\left(N-n_{2}\right)\\ c_{2}=c_{1}\cdot n_{3}/\left(N-n_{3}\right) \end{array}\right.$$

**Figure 3:**
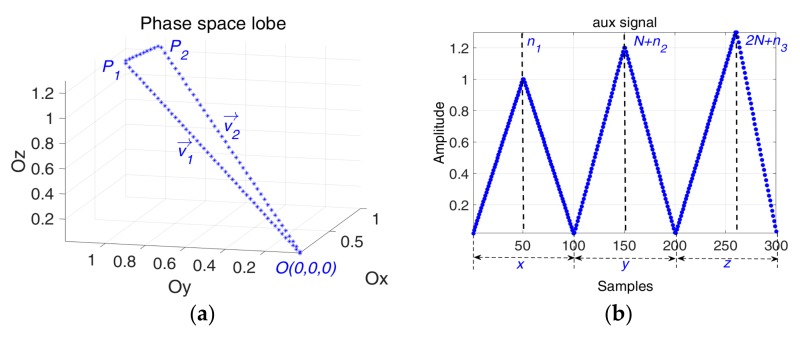
(**a**) The generated phase space lobe; (**b**) The corresponding aux signal based on Equations (5) and (7) with the following parameters: N=100 , $a_{1}=1,\;\;b_{1}=1.2,\;\;c_{1}=1.3$, $n_{1}=50,\;\;n_{2}=50,\;\;n_{3}=60$.

**Figure 4:**
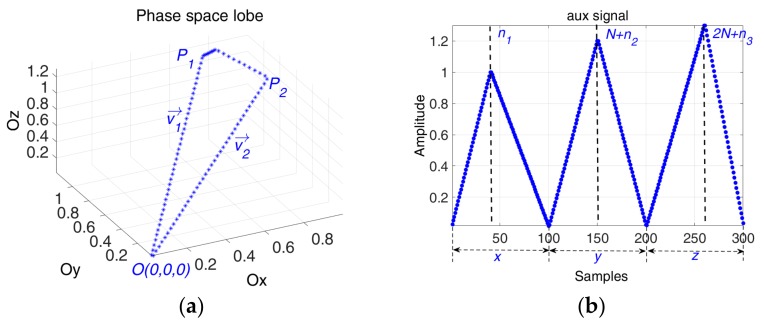
(**a**) The generated phase space lobe; (**b**) The corresponding aux signal based on Equations (5) and (7) with the following parameters: N=100 , $a_{1}=1,\;\;b_{1}=1.2,\;\;c_{1}=1.3$, $\;n_{1}=40,\;\;n_{2}=50,\;\;n_{3}=60$.

Figure 3 and Figure 4 show that different segments of the trajectory appear when the interval defined by $n_{1},\;\;n_{2}$ and $n_{3}$ are not coincident.

**Figure 5:**
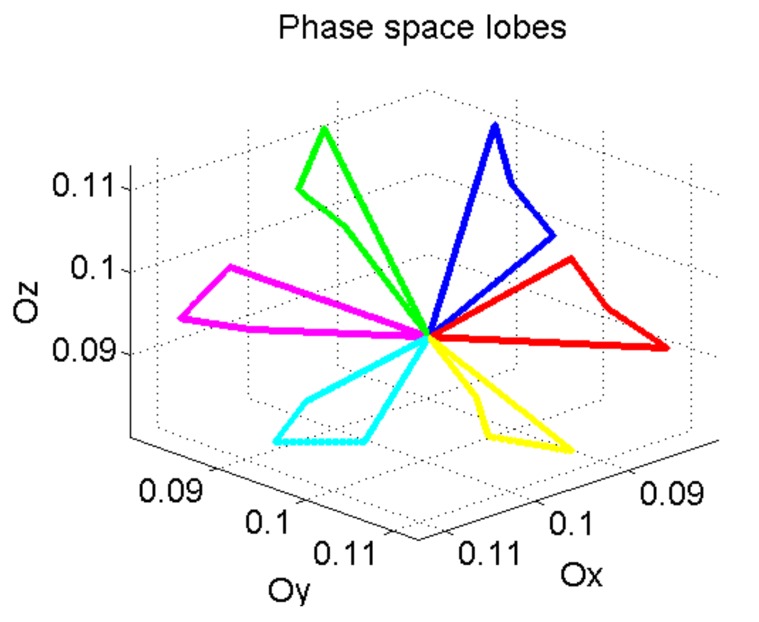
Phase space lobes generated from the set of 6 auxiliary signals used as IF laws for the signals defined in time domain.

Using these characteristics in different combinations, we get separable phase diagram lobes (Figure 5). This auxiliary signal (phase space lobe) is used as frequency modulating signal in order to be recovered at the receiving level and separated from other lobes in the phase diagram.

### 3.3. Phase Diagram “Marked” Lobes for Emission

This new waveform generation technique is based on the information coding via a frequency modulation defined by the lobes designed in the phase space. If the signals defined from these lobes are used in a time-of-flight (TOF) measurement application, we introduce a special marker that will help measuring the time of flight as the time distance between these markers observed in the phase space.

**Figure 6:**
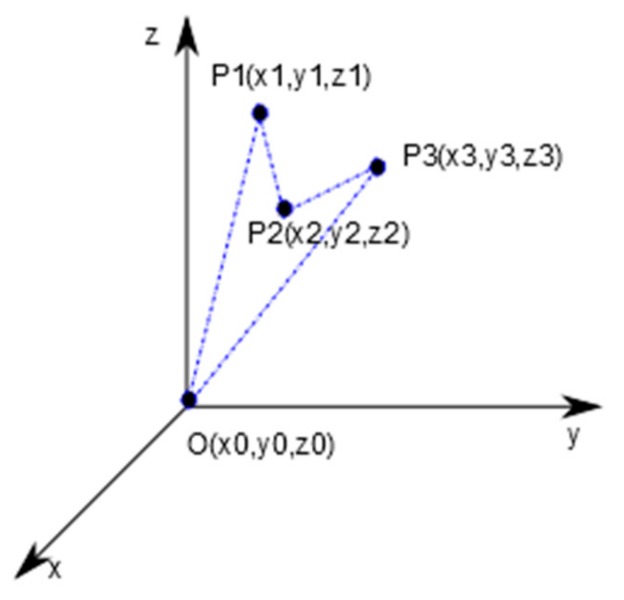
The ideal phase space representation of the “marked” lobe (auxiliary signal).

In this case, the two director vectors from Equation (5) define the lobe in the Cartesian system which presents an extra point as the marker of the lobe highlighted in Figure 6.

**Figure 7:**
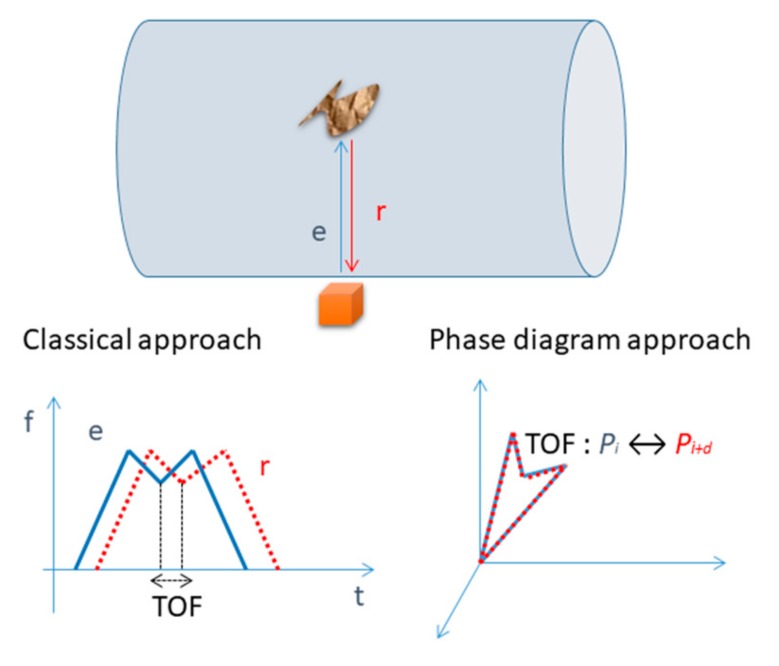
TOF estimation: classical approach versus phase diagram approach; the estimation of TOF is done using the delayed samples between the marks of emitted and received signals, respectively.

The “signature” of the lobe is given by the point $P_{2}(x_{2},y_{2},z_{2})$ in the phase space (Figure 6). Our proposal to add an extra point is given by the fact that another mark would decrease the estimation error in the phase diagram. Based on this “signature”, we aim to determine the TOF (Figure 7). The interest of this approach is that, even if the IF laws between transmitter and the receiver are not the same (due to propagation effect or event estimation error), if the markers are still visible, they allow a more accurate estimation of TOF than looking in time domain.

The points obtained on the phase diagram corresponding to the positions of $P_{1}$, $P_{2}$ and $P_{3}$ (shown in Figure 6) have a certain delay, $\delta_{i}$, between the emitted signal and the received one. Therefore, the estimated TOF corresponds to the estimated delay, $\hat{\delta }$ given by Equation (8):

(8) $$\hat{\delta }=\sum_{i}\frac{\delta_{i}}{p},i=\{1,2,3\}$$

where $\delta_{i}$ is the delay for each “signature” point $P_{i}$ between the emission and reception signal.

With this approach, the delay estimation is achieved with smaller error than in the case of time-frequency representation which is sensitive to the time-frequency resolution and noise presence.

The first condition which we imposed is: the four points $O,P_{1},P_{2}$ and $P_{3}$ should be coplanar. It goes that:

(9) $$\left|\begin{array}{cccc} x_{0} & y_{0} & z_{0} & 1\\ x_{1} & y_{1} & z_{1} & 1\\ x_{2} & y_{2} & z_{2} & 1\\ x_{3} & y_{3} & z_{3} & 1 \end{array}\right|=0$$

Considering $x_{0}=y_{0}=z_{0}=0$, Equation (9) is equivalent to:

(10) $$\left|\begin{array}{ccc} x_{1} & y_{1} & z_{1}\\ x_{2} & y_{2} & z_{2}\\ x_{3} & y_{3} & z_{3} \end{array}\right|=0$$

It is obvious that the points *P*_1_, *P*_2_ and *P*_3_ should be different, therefore the lines of the determinant from Equation (10) must be different.

In order to accomplish the condition of coplanarity for whatever points $P_{1},P_{2}$ and $P_{3}$, at least two columns of the determinant should be proportional. Hereby, the coplanarity condition (Equation (9)) restraints to the second condition:

(11) $$\frac{x_{i}}{z_{i}}=r,\;\;i\in \{1,2,3\},\;\;r\in (1,2)$$

As third condition, the second column is defined as:

(12) $$y_{1}=x_{3},y_{2}=x_{2}\;\;\mathrm{and}\;\;y_{3}=x_{1}$$

With this condition, the phase space representation forms a lobe with a “signature” provided that $x_{1}>x_{2}\;$ and $\;x_{3}>x_{2}$.

Next, the signals are generated in the same way as in the case of the simple lobe: the IF laws (auxiliary signals) are used to obtain the frequency modulated wide band signals.

**Figure 8:**
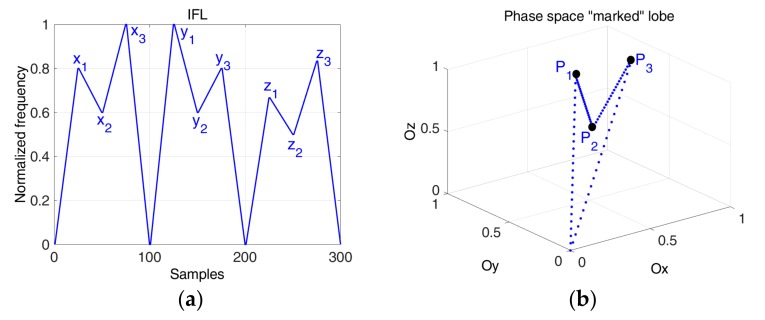
(**a**) The auxiliary signal; (**b**) The phase space marked lobe: $x_{1}=0.8,\;\;x_{2}=0.6,x_{3}=1\;$ ,  r=1.2.

**Figure 9:**
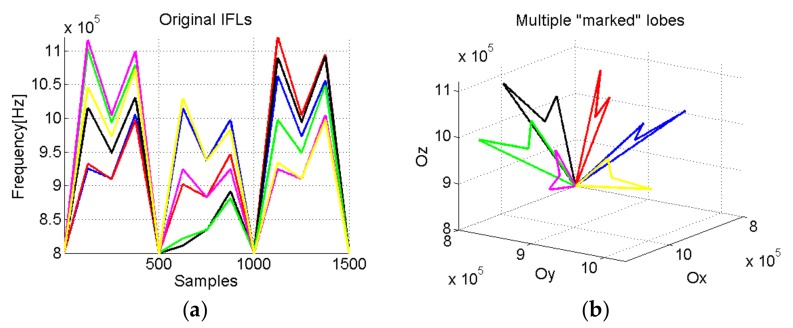
(**a**) IF law auxiliary multiple signals; (**b**) The multiple lobes with signature obtained based on the lobe from Figure 8 and rotated with an angle of π/6.

The multiple lobes approach results from the rotation in the phase space, with a given angle, of one “marked” lobe (for example, the one from Figure 8). In this way, we generate multiple “marked” lobes, as shown in Figure 9.

## 4. Phase Diagram RQA Based IF Law Estimation

In this section, we present a new technique for IF law estimation based on the phase diagram representation and the recurrence quantification analysis. This approach is, once again, suggested by the evolution of a signal in the phase diagram and its conservative properties presented in [15,16], namely the recurrence of states [27,28,29].

### 4.1. Signal’s Characteristics in the Phase Diagram Representation

The main attributes of this representation are highlighted by considering three signals $s_{1}$, $s_{2}$ and $s_{3}$, which depend on a signal s as follows:

(13) $$s_{1}[n]=s[n+\delta ]$$

(14) $$s_{2}[n]=s[\alpha n]$$

(15) $$s_{3}[n]=\beta \cdot s[n]$$

where δ,  α and β are constants that modify the s signal through translation in time, scale and amplitude, respectively. Then, phase-space points present the following attributes [2,18,30]:

(16) $$\vec{v_{1[i]}}=\vec{v_{\left[i+\delta \right]}}$$

(17) $$\vec{v_{2[i]}}=\vec{v_{\left[\alpha i\right]}}$$

(18) $$\vec{v_{3[i]}}=\beta \cdot \vec{v_{\left[i\right]}}$$

The phase diagram trajectory is invariant to translation in time, Equations (13) and (16). In terms of scale and amplitude changes, from Equations (14) and (17), the trajectory of the phase diagram preserves the properties of the signals, the $\vec{v_{2}}$ trajectory is “drawn” α times faster than the $\vec{v}$ trajectory, respectively, the $\vec{v_{3}}$ components are β times larger than the $\vec{v}$ components, Equation (18).

**Figure 10:**
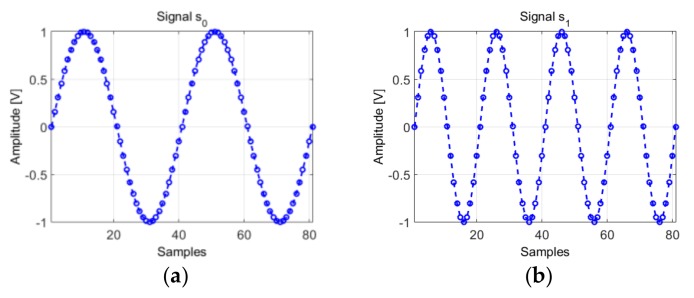
(**a**) The generated signal $s_{0}$ from Equation (19); (**b**) The generated signal $s_{1}$ from Equation (19).

The information contained in the scale changes, Equation (14) remain available in the phase diagram trajectory, Equation (17) and are exploited in the phase diagram RQA based IF law tracking. This effect is described for the following signals shown in Figure 10:

(19) $$\begin{array}{l} s_{0}[n]=\sin\left(2\pi f_{0}\frac{n}{f_{s}}\right),\;\;n\in \{0,1,\ldots ,\frac{2f_{s}}{f_{0}}\}\\ s_{1}[n]=\sin\left(2\pi f_{1}\frac{n}{f_{s}}\right),\;\;\;f_{1}=2f_{0},\;\;n\in \{0,1,\ldots ,\frac{2f_{s}}{f_{0}}\} \end{array}$$

where $f_{0}=10$ and $f_{s}=400$.

**Figure 11:**
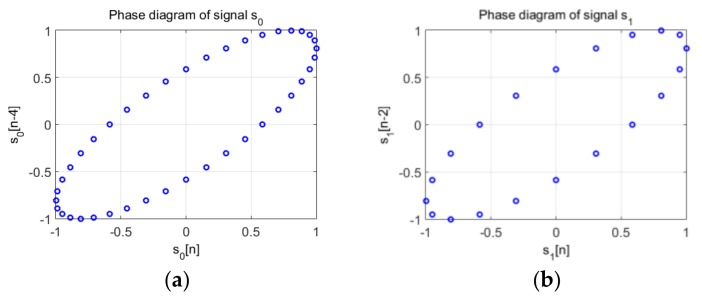
(**a**) The phase space trajectory of the signal $s_{0}$ from Equation (19) where d=4 and m=2; (**b**) The phase space trajectory of the signal $s_{1}$ from Equation (19) d=2 and m=2.

Figure 11 presents the trajectory in the phase diagram of the signals considered in Equation (19) both sine waves are represented by an ellipse with the same parameters: these characteristics are obtained by considering $d_{0}=2d_{1}$, because $f_{1}=2f_{0}$.

Moreover, given that both signals have the same sampling period, the ellipse corresponding to the trajectory of $s_{1}$ is “drawn” two times faster than the ellipse corresponding to signal $s_{0}$. In other words, the points on the trajectory are plotted at the same “speed” (because the sampling frequency is the same), therefore, the fundamental period of the signal results from a complete rotation of the ellipse described by the position vectors of the trajectory, $\vec{v_{0[i]}}$, and respectively $\vec{v_{1[i]}}$. It takes two complete rotations in the phase diagram of the position vector to describe the trajectory of the signal $s_{1}$, while one complete rotation describes the trajectory of signal $s_{0}$.

Following the observations presented above, in the case of the phase space RQA based IF law estimation, the below algorithm has been established:

- The trajectory of the analyzed signal is computed in the phase diagram for the embedding dimension m=2 (previously, the signal can be interpolated)
- The angular distance between successive points of the phase diagram is applied, namely the polar angle of each position vector $\vec{v_{\left[n\right]}}$ is computed as follows:
  (20) $$\theta [n]=\arctan\left(\frac{x[n+d]}{x[n]}\right)$$
- Next, the ellipse’s evolution is quantified as follows: a counter is started when the first point of the trajectory has a negative polar angle and is followed by point with a positive polar angle on the trajectory. This starting point marks the starting of a half of rotation. The value of the counter is increased successively until the angle passes again from positive to negative. This means that half of rotation of the trajectory has been reached and the angular distance between the starting point and the current point is π. Let this number be $N_{k_{1}}$. The position of this point is diametrically opposed to the starting point of the counter in the phase diagram.
- The algorithm continues with the next round of points until the next π angular distance is obtained. Let $N_{k_{2}}$ be the numbers of points contained in this region.
- With the number of points counted in these two successive intervals, $n_{0}=N_{k_{1}}+N_{k_{2}}$, we obtain the first estimation, $f_{0}$, where $f_{0}=\frac{f_{s}}{n_{0}}$.
- The algorithm continues until the last rotation of the trajectory from the phase diagram is reached.

Given the fact that dynamics of a nonlinear system may rapidly change, in the next subsection, we compute the phase diagram RQA based IF law estimation only for a half of rotation.

### 4.2. IF Law Estimation Using the Phase Diagram RQA Approach

**Figure 12:**
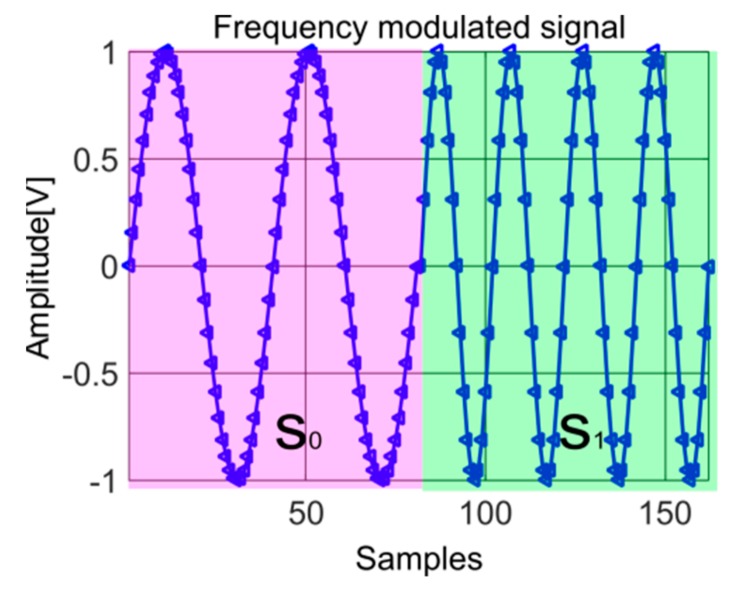
Signal with two successive frequencies, s, used for the illustration of the IF law estimation method.

This subsection presents the algorithms for the phase diagram RQA IF law estimation concept. For illustration purposes, let us consider a two tone signal, s, Equation (21), with two successive frequency variations plotted in Figure 12:

(21) $$s=\left\{\begin{array}{l} s_{0}=\sin\left(2\pi f_{0}\frac{n}{f_{s}}\right),\;\;n\in \{0,1,\ldots ,\left[\frac{2f_{s}}{f_{0}}\right]\}\\ s_{1}=\sin\left(2\pi f_{1}\frac{n}{f_{s}}\right),\;f_{1}=2f_{0},\;\;\\ \;\;\;\;\;\;\;\;\;\;\;\;\;\;\;\;\;\;\;\;\;\;\;\;\;\;\;\;\;\;\;\;\;\;\;\;\;\;\;\;\;\;\;\;n\in \{\left[\frac{2f_{s}}{f_{0}}\right]+1,\left[\frac{2f_{s}}{f_{0}}\right]+2,\ldots ,\left[\frac{2f_{s}}{f_{0}}\right]\} \end{array}\right.$$

where $\left[x\right]$ is the integer part of x.

**Figure 13:**
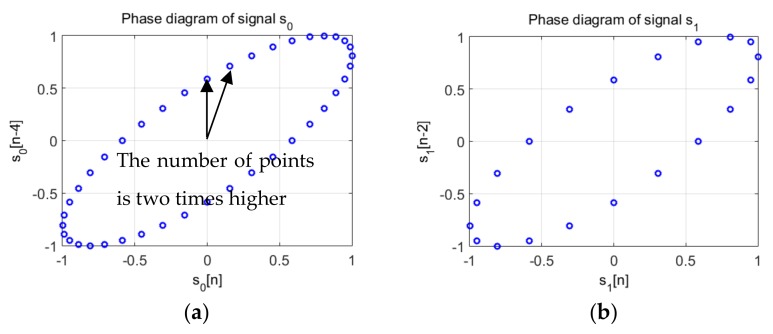
(**a**) The phase space trajectory of the signal $s_{0}$ from Equation (19); (**b**) The phase space trajectory of the signal $s_{1}$ from Equation (19); The number of samples of each trajectory is inversely proportional with the frequency.

Figure 13 shows the phase diagrams for the two parts of the signal of frequency $f_{0}$ (the lower one) and $f_{1}$ (the higher one). As this figure shows, the trajectories are the same, but the number of points of the trajectory are related to the frequencies. For the $f_{0}$ frequency, twice lower than $f_{1}$, the number of points is double compared to the phase space representation of the higher frequency, $f_{1}$.

In order to automatically quantify the number of recurrent points from the trajectory, directly related to the instantaneous frequency, we firstly compute, for each pair of points, the angle between two position vectors, namely their angular distance as:

(22) $$\alpha_{i}=\arccos\left(\frac{\vec{v_{\left[i\right]}}\cdot \vec{v_{\left[i+1\right]}}}{\left|\vec{v_{\left[i\right]}}\right|\cdot \left|\vec{v_{\left[i+1\right]}}\right|}\right)$$

With these values, we can quantify the number of points in the trajectory according to the condition (23) that the angular distance has to meet:

(23) $$N_{k}\;\;is\;\;given\;\;by\sum_{j=i}^{i+N_{k}-1}\alpha_{j}=\pi$$

The instantaneous frequency is then estimated as according to Equation (24):

(24) $${\hat{f}}_{k}=\frac{f_{s}}{2N_{k}}$$

The estimation of the IF law is obtained from the following expression:

(25) $$IFL(k)=\frac{f_{s}}{2N_{k}},\;\;\mathrm{where}\;\;N_{k}\left|\sum_{j=i}^{i+N_{k}-1}\alpha_{j}=\pi \right.$$

Still, the choice of the delay d must be discussed. The optimal delay depends on the signal’s characteristics; we propose the use of the multi-lag phase analysis (MLPA) [16,28,29] for the IF law estimation.

**Figure 14:**
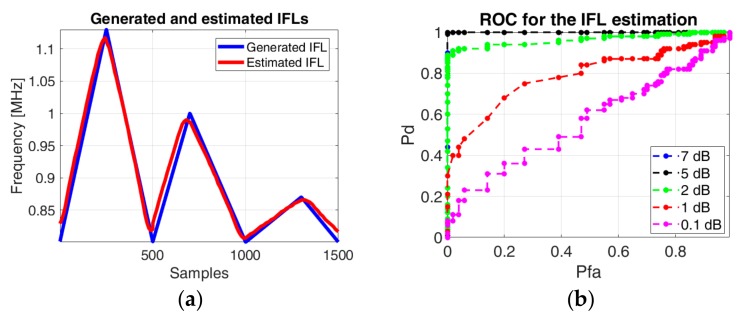
(**a**) The IF law used for generation (in blue) and the phase diagram based estimated IF law (in red); (**b**) The receiver operating characteristic (ROC) for the IF law estimation.

Based on this approach, we present the IF law estimation results for a signal, $s_{IFL}$:

(26) $$\begin{array}{l} s_{IFL}=\sin(\phi (t)),\;\;t\in [0,1.5\mu s]\\ IFL=\frac{1}{2\pi }\frac{\partial \phi (t)}{\partial t} \end{array}$$

where ϕ is the phase of the signal and the IFL parameter varies according to the blue line from Figure 14.

For the signal considered for estimation, we applied additive white Gaussian noise on the generated signal, so that the ratio between energy of the generated signal and the energy of the noise is SNR = 7 dB (Figure 14a). The estimated IF law is plotted as a red curve, showing an accurate estimation in the given conditions.

Tests regarding the IF law estimation for different SNRs have been performed in order to emphasize the applicability of the method on real signals (Figure 14b). For a SNR above 5 dB, the method succeeds to estimate the IF law accurately.

## 5. High Speed Sensing Using Phase Diagram-Based Adaptive Waveform

In this section, we illustrate the concept of the high speed sensing using the phase-diagram-based adaptive waveform. Namely, we illustrate the capacity of this concept to handle the overlapped IF laws. We also show the interest of marked lobes to accurately estimate the time-of-arrival (TOA) in an acoustic sensing system.

### 5.1. Partially Overlapped IF Laws at Reception

**Figure 15:**
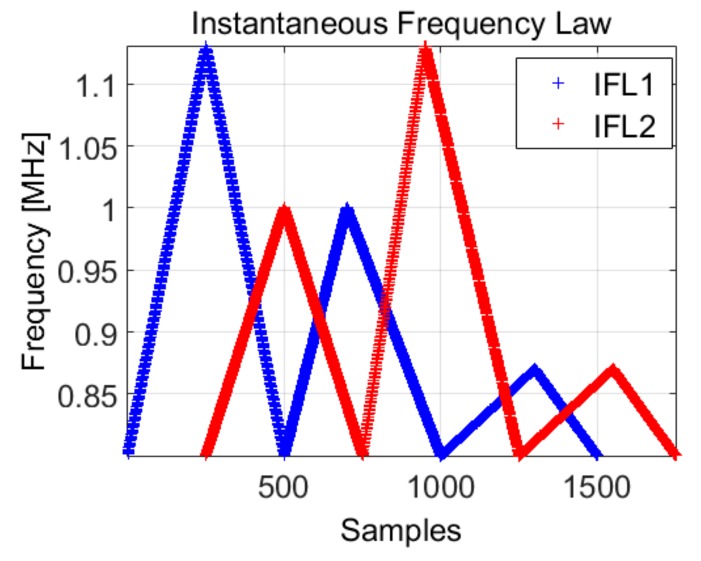
The IF laws considered for emission in Equation (27) for high speed sensing in dynamic environments.

In this example, we consider the case of a signal composed of two frequency modulated components. The theoretical IF law is plotted in Figure 15.

These signals which are partially overlapped are suited for high speed sensing in dynamic phenomena, because they can be sent with a pulse-repetition-frequency (PRF) much lower than in a classical radar context, where the PRF is defined by the maximal sensing range:

(27) sIFL12=s1(t)+s2(t)                =ReAejϕ1(t)+ejϕ2(t)ϕ1(t)=2π∫0t1IFL1(t)dtϕ2(t)=2π∫t0t2IFL2(t)dt

where the IFL1 overlaps IFL2, but it is shifted in time with 0.25 μs.

The IFL1 has the following parameters $a_{1}=1.13\;\mathrm{MHz},\;\;b_{1}=1\;\mathrm{MHz},$$\;c_{1}=0.87\;\mathrm{MHz}$, $n_{1}=0.5$, $n_{2}=0.4$ and $n_{3}=0.6$, respectively IFL2 is characterized by $a_{1}=1\;\mathrm{MHz},$ $b_{1}=1.13\;\mathrm{MHz},$ $c_{1}=0.87\;\mathrm{MHz}$, $n_{1}=0.5$, $n_{2}=0.4$ and $n_{3}=0.6$. Both signals have the duration of 50 μs and the sampling frequency of 10  MHz, hereby N=500.

**Figure 16:**
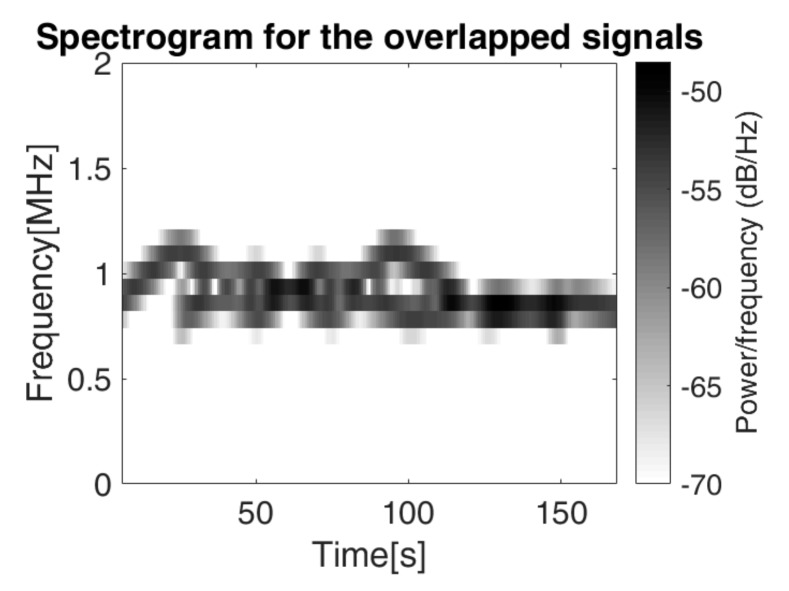
The spectrogram of the signal *s_IFL_*_12_ containing two overlapped IF laws computed for a window of 128 samples, 120 overlapping samples and 128 points for the Fourier transform computation.

The spectrogram of this signal is represented in Figure 16, for a SNR = 7 dB, making it difficult to separate between the two IF laws. We recall that for the overlapped signals considered for estimation, we also applied additive white Gaussian noise, so that the ratio between energy of the generated signal and the energy of the noise is SNR.

**Figure 17:**
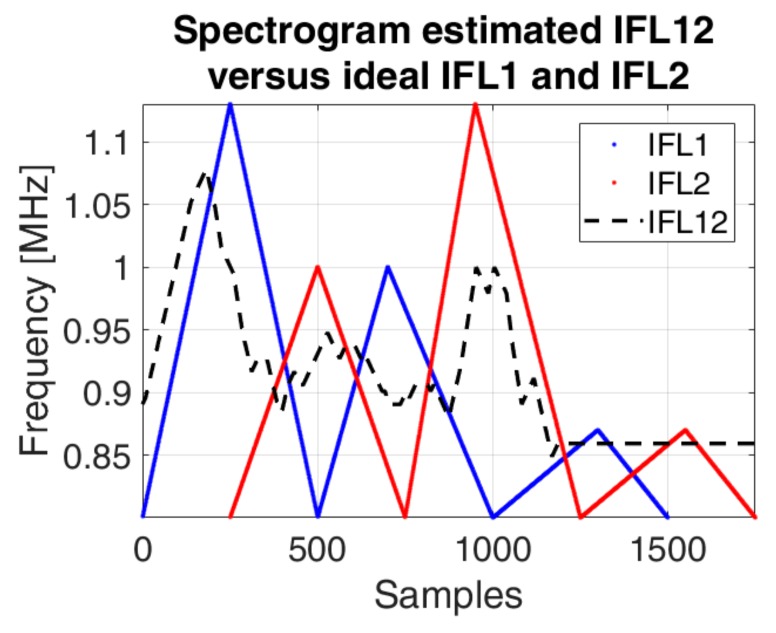
The spectrogram based estimated IF law of the signal *s_IFL_*_12_ (dashed black) versus the ideal IF laws: *IFL*1 (blue) and *IFL*2 (red).

**Figure 18:**
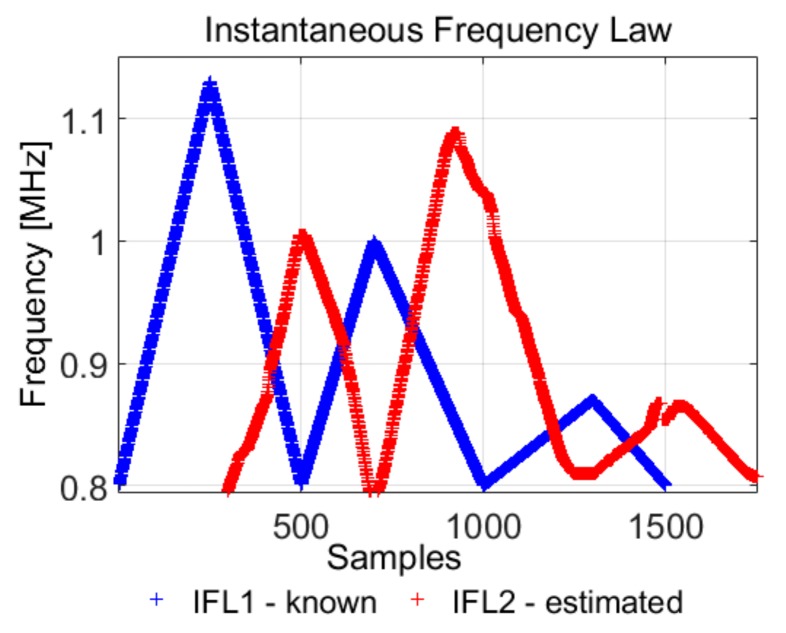
The phase space based estimation of the overlapped IF laws: *IFL*1 is known, *IFL*2 is estimated.

**Figure 19:**
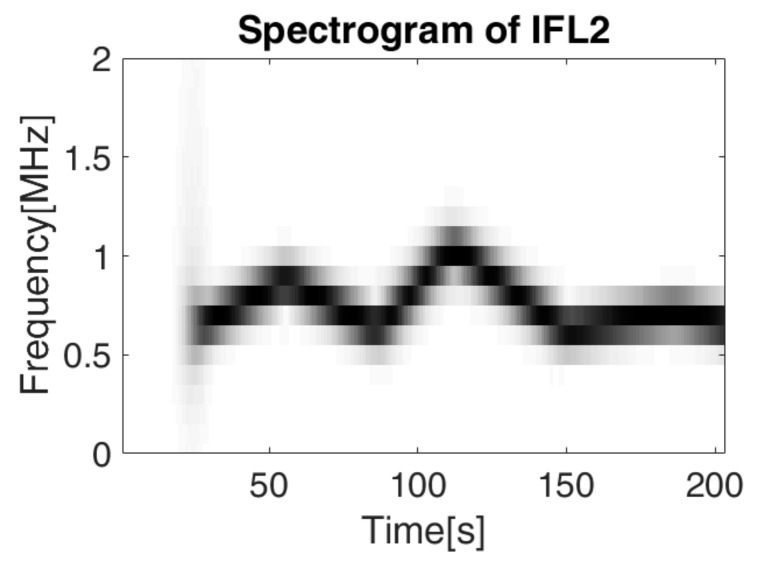
The estimation of the overlapped IF laws using the spectrogram: *IFL*2 is estimated by subtracting *IFL*1 (for a window of 128 samples, 120 overlapping samples and 128 points for the Fourier transform computation).

The spectrogram approach does not provide a reliable method for the separation of the two IF laws, because it is not able to distinguish between the time overlapped frequencies (especially for the low variations), it just determines the resulting instantaneous frequency. Considering the algorithm presented in Section 4.2. and the first IF law (*IFL*1) known, we intend to separate the second IF law (*IFL*2) from the first. The results are presented in Figure 17, Figure 18 and Figure 19.

Although the IFL2 is also computed with the spectrogram, the estimation is difficult for small variations, whereas the IF law estimation using the phase diagram of each component is possible due to the natural continuity property of the phase diagram: each IF laws will continuously evaluate in its way, without interacting with the other components. Using Equation (25), the instantaneous estimated frequency is, in fact, the sum of the two frequencies given by *IFL*1 and *IFL*2.

### 5.2. “Marked Lobes” for Emission and Reception

This approach can be used in the case of active sensing, in order to detect objects (such as cavitating bubbles and oil pockets). The detection and estimation of object properties are based on the received signal’s parameters like TOFs or Doppler deformation.

Generally, current sensing techniques are able to provide these parameters just for few periods. While this might be enough for the characterization of the phenomena, there is always need for a more detailed understanding of these phenomena. Hence, the estimation of the signal’s parameters for a large number of periods is necessary. This is actually what we call high speed sensing and this subsection presents the feasibility of this concept using the phase diagram-based adaptive waveform.

**Figure 20:**
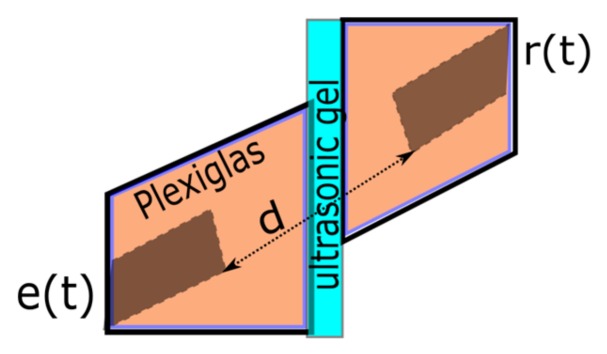
The positioning of the two transducers for the experimental test.

**Figure 21:**
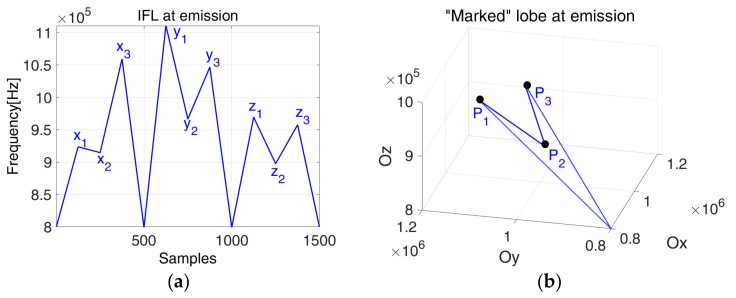
(**a**) The IF law used at emission; (**b**) the corresponding “marked” phase space lobe.

The experimental aspect is an essential part with respect to possible applications of the concept in real–life, therefore we considered placing of the ultrasonic piezoelectric transducers as shown in Figure 20. Figure 21 presents the considered IF law and its corresponding phase space lobe used as emission signal, e(t).

The central frequency of the transducers is 1 MHz and their bandwidth is around 400 kHz. The ultrasonic gel thickness is under 1 mm. The transducers are mounted within a Plexiglas support and the measured distance between them is *d* = 4 cm. The placement of the sensors corresponds to $TOF_{real}=23.529\;\mu s$, considering the speed of sound in Plexiglas v=1700   m/s.

**Figure 22:**
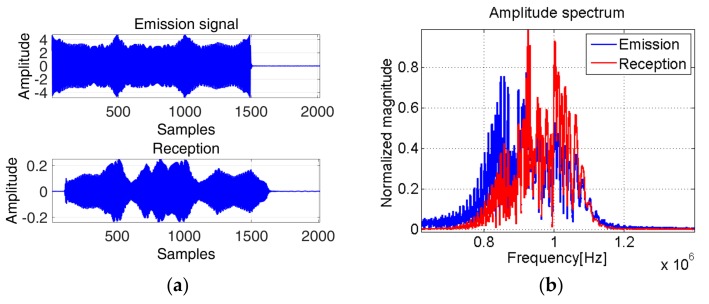
(**a**) The emitted and received signal in time; (**b**) The corresponding amplitude spectrum.

The test consists in verifying if the characteristics of the signal containing this IF law remain unchanged when using two ultrasonic transducers for emission and, respectively, for reception. With this, we aim to estimate the TOF between the transducers. The two transducers are placed one in front of the other within their Plexiglas support. The signals used for emission and reception are shown in the next figure, Figure 22.

**Figure 23:**
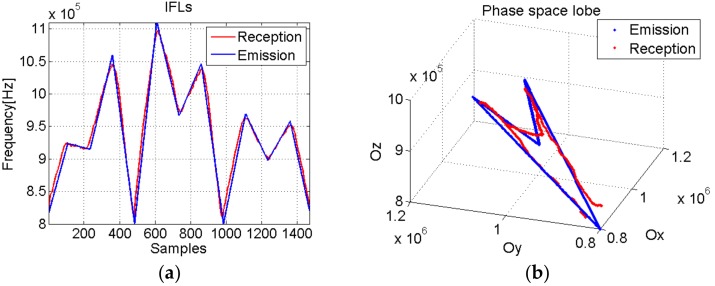
(**a**) The IF laws of the emitted and received signals; (**b**) The phase space representation of the IF laws.

Figure 23 shows that although the transfer characteristic of the transducers eliminates part of the lower frequencies, the received signal maintains our proposed characteristics. Therefore, these new signals with “special signatures” in the phase diagram representation preserve their properties to be explored in real-life applications.

With the use of the spectrogram (computed on a window of 64 samples, a number of 62 samples of overlap between adjoining segments and 64 points for the computation of the discrete Fourier transform), we have obtained the TOF between the emitted and received signal $TOF_{spec}=20\;\mu s$.

Based on the phase diagram analysis, the delay between the emitted and received signal δ=112 which corresponds to $TOF_{ph\_diag}=22.4\;\mu s$.

The TOF estimation results obtained using the phase diagram approach are closer to $TOF_{real}$, than the spectrogram approach. In terms of relative error, the spectrogram approach presents an error of 15%, whereas the phase diagram approach presents an error of 4.8%.

## 6. Conclusions

The phase diagram is a powerful concept derived from nonlinear dynamic systems theory and presents the major advantage to characterize the trajectory of the system without any a priori information about the system meaning that there is no assumption made about the system and its propagation environment before analyzing the data.

Considering a non-stationary signal, it is assumed that the dynamic evolution of the system is preserved in the phase diagram representation, therefore the recurrent points of the phase space are quantified and related in time to the fundamental frequency of the signal. Hereby, two new concepts have been defined.

The first one deals with the adaptive waveform design using separable lobes defined in the phase diagram domain. Different regions of the phase diagram are used to define these lobes. Lobes transformation in frequency modulation vectors leads to the design of a set of frequency modulated signals, non-orthogonal in the time/frequency domain, but separable in the phase diagram domain. This property helps us defining a high speed sensing system since we are able to transmit, in very short time slots, different wide-band waveforms. In addition, the marked lobes will help improving the time-of-arrival (TOA) estimation.

At the receiving level, in order to perform the estimation of TOAs, we have developed an algorithm for the estimation of the IF law of a signal. Knowing that a sine wave’s trajectory in the phase diagram is an ellipse, the concept of IF law tracking is the following: once the system returns in a previously visited point on the trajectory (a recurrent state)—namely the position vector of the trajectory has performed a complete rotation of the ellipse—the speed of rotation of the position vector is directly related to the local frequency of the signal.

The concept of high speed sensing allows us to get the dynamic evolution of a process or an object’s image. For this purpose, the phase diagram-based waveform design can be successfully used, as illustrated in the final part of Section 5. Namely, we have shown that the estimation of the TOFs by phase diagram-based approach has an error below 5% whereas this error is of 15% using the spectrogram-based technique. This relative good accuracy comes from the data representation in phase diagram domain, allowing a better separation between the transmitted components as well as the capability of IF law estimation in this domain.

The concept proposed for the receiver part, allows us estimating the IF law, without any required model and/or an initial representation of the signal in time-frequency domain. The non-parametric and automatic computation of IF laws of the signal allows to imagine a large range of application, such us underwater signals analysis, analysis of communication signals, analysis of acoustic and electromagnetic transients, etc. Generally speaking, this approach can successfully be applied in signal analysis in active or passive context, for detection and classification purposes. As an example, we illustrate the results of the IF law estimation for two communication signals, where we can observe how the differences between the two signals can be easily highlighted.

Other applications of this approach, related to the underwater signal analysis and the classification of transients in power networks, are now in progress. Further developments will focus on investigating the capability of instantaneous frequency law tracking in the phase diagram, in the presence of many components, trying to take advantage of the phase continuity of the phase trajectory. Namely, the IF law tracking can be successfully achieved using the association of the time-frequency points according to the continuity criteria that could be efficiently exploited in the phase diagram domain. In the case of the multi-lag phase diagram analysis, this continuity will be defined in a more robust manner since the random noise effect will be minimized when looking to different lags.

## References

[1] Digulescu A. (2017). Characterization of Dynamic Phenomena Based on the Signal Analysis in Phase Diagram Representation Domain. Ph.D. Thesis.

[2] Digulescu A., Vasile C., Ioana C., Serbanescu A., Vasile G., Mars J. Instantaneous frequency law tracking using signal’s representation in phase diagram domain. Proceedings of the 2017 22nd International Conference on Digital Signal Processing (DSP).

[3] Yuan C., Azimi-Sadjadi M., Wilbur J., Dobeck G. (2000). Underwater target detection using multichannel subband adaptive filtering and high-order correlation schemes. IEEE J. Ocean. Eng..

[4] Papandreou-Suppappola A., Muray R., Iem B.G., Boudreaux-Bartels G.F. (2011). Group delay shift covariant quadratic time-frequency representations. IEEE Trans. Signal Process..

[5] Djurović I., Stanković L.J. (2004). An algorithm for the Wigner distribution based instantaneous frequency estimation in a high noise environment. Signal Process..

[6] Cakrak F., Loughlin P.J. (2001). Multiple window time-varying spectral analysis. IEEE Trans. Signal Process..

[7] Barkat B., Boashash B. (1999). Instantaneous frequency estimation of polynomial FM using the peak of the PWVD: Statistical performance in the presence of additive Gaussian noise. IEEE Trans. Signal Process..

[8] Boashash B., O’Shea P. (1994). Polynomial wigner-ville distributions and their relationship to time-varying higher order spectra. IEEE Trans. Signal Process..

[9] Stanković L.J., Djurović I., Ohsumi A., Ijima H. Instantaneous frequency estimation by using wigner distribution and viterbi algorithm. Proceedings of the 2003 IEEE International Conference on Acoustics, Speech, and Signal Processing.

[10] Abdoush Y., Garcia-Molina J.A., Corazza G.E. (2019). Adaptive instantaneous frequency estimation based on time-frequency distributions with derivative approximation. Signal Process..

[11] Hussain Z.M., Boashash B. Adaptive instantaneous frequency estimation of multi-component FM signals. Proceedings of the 2000 IEEE International Conference on Acoustics, Speech and Signal Processing. Proceedings (Cat. No.00CH37100).

[12] Lerga J., Sucic V. (2009). Nonlinear IF estimation based on the pseudo WVD adapted using the improved sliding pairwise ICI rule. IEEE Signal Process. Lett..

[13] Zbilut J., Webber C. (1992). Embeddings and delays as derived from quantification of recurrence plots. Phys. Lett. A.

[14] Webber C., Zbilut J. (1994). Dynamical assessment of physiological systems and states using recurrence plot strategies. J. Appl. Physiol..

[15] Webber C., Zbilut J. (2005). Recurrence quantification analysis of nonlinear dynamical systems. Tutorials in Contemporary Nonlinear Methods for the Behavioral Sciences Web Book.

[16] Marwan N. (2003). Encounters with Neighbours—Current Developments of Concepts Based on Recurrence Plots and Their Applications. Ph.D. Thesis.

[17] Ioana C., Digulescu A., Serbanescu A., Candel I., Birleanu F. (2014). Recent advances in non-stationary signal processing based on Recurrence Plot Analysis concept. Translational Recurrences. From Mathematical Theory to Real-World Applications.

[18] Birleanu F.-M., Ioana C., Gervaise C., Chanussot J., Serbanescu A., Serban G. On the recurrence plot analysis method behaviour under scaling transform. Proceedings of the 2011 IEEE Statistical Signal Processing Workshop (SSP).

[19] Mallat S. (1999). A Wavelet Tour of Signal Processing.

[20] Mallat S., Zhong S. (1992). Characterization of signals from multiscale edges. IEEE Trans. Pattern Anal. Mach. Intell..

[21] Eckmann J., Kamphorst S., Ruelle D. (1987). Recurrence plots of dynamical systems. Europhys. Lett..

[22] Kantz H., Schreiber T. (1997). Nonlinear Time Series Analysis.

[23] Marwan N., Romano M., Thiel M., Kurths J. (2007). Recurrence plots for the analysis of complex systems. Phys. Rep..

[24] Webber C. (2013). Recurrence quantification of fractal structures. Front. Physiol..

[25] Zbilut J., Webber C. (2006). Recurrence Quantification Analysis.

[26] Komalapriya C., Thiel M., Romano M.C., Marwan N., Schwarz U., Kurths J. (2008). Reconstruction of a system’s dynamics from short trajectories. Phys. Rev. E.

[27] Marwan N. (2008). A Historical review of recurrence plots. Eur. Phys. J. Spec. Top..

[28] Bernard C. (December 2015). Characterization of Physical Phenomena Using Parsimonious Analysis of Transients Signals. Ph.D. Thesis.

[29] Marwan N., Schinkel S., Kurths J. (2013). Recurrence plots 25 years later–Gaining confidence in dynamic transitions. Europhys. Lett..

[30] Bernard C., Petrut T., Vasile G., Ioana C. Multi-lag phase space representations for transient signal characterization. Proceedings of the 2014 22nd Europeean Signal Processing Conference (EUSIPCO).

